# Effect of oligonucleotide primers in determining viral variability within hosts

**DOI:** 10.1186/1743-422X-1-13

**Published:** 2004-12-09

**Authors:** Maria Alma Bracho, Inmaculada García-Robles, Nuria Jiménez, Manuela Torres-Puente, Andrés Moya, Fernando González-Candelas

**Affiliations:** 1 Institut *Cavanilles *de Biodiversitat i Biologia Evolutiva, Universitat de València, Edifici Instituts, Polígon "La Coma" s/n, Paterna (València) 46980 SPAIN

## Abstract

**Background:**

Genetic variability in viral populations is usually estimated by means of polymerase chain reaction (PCR) based methods in which the relative abundance of each amplicon is assumed to be proportional to the frequency of the corresponding template in the initial sample. Although bias in template-to-product ratios has been described before, its relevance in describing viral genetic variability at the intrapatient level has not been fully assessed yet.

**Results:**

To investigate the role of oligonucleotide design in estimating viral variability within hosts, genetic diversity in hepatitis C virus (HCV) populations from eight infected patients was characterised by two parallel PCR amplifications performed with two slightly different sets of primers, followed by cloning and sequencing (mean = 89 cloned sequences per patient). Population genetics analyses of viral populations recovered by pairs of amplifications revealed that in seven patients statistically significant differences were detected between populations sampled with different set of primers.

**Conclusions:**

Genetic variability analyses demonstrates that PCR selection due to the choice of primers, differing in their degeneracy degree at some nucleotide positions, can eclipse totally or partially viral variants, hence yielding significant different estimates of viral variability within a single patient and therefore eventually producing quite different qualitative and quantitative descriptions of viral populations within each host.

## Background

One of the most difficult tasks faced by virologists is the documentation and evaluation of the genetic variability of viral populations in infected patients. These analyses are greatly facilitated by the use of the polymerase chain reaction (PCR). PCR based techniques do not always produce a highly specific and homogeneous product. When the template is a complex mixture of homologous sequences the aim of the amplification would be to preserve as much as possible the template-to-product ratios of every sequence in order to obtain a good representation of the diversity present in the initial sample. PCR products are derived from templates by a process involving complex chemical kinetics, and the relative abundance of the different homologous genomes among the final products is often a parameter of interest. This is the case, for instance, in experiments aimed at determining natural diversity in microbial communities [1] or at identifying members of multigene families [2] and it is of special relevance for studies of viral variability within hosts, especially for highly variable RNA viruses.

The precise mechanisms involved in the preferential amplification of some templates from non-homogeneous sources are not fully understood and should be differentiated from those related to stochastic or tube-to-tube variations in amplification efficiency. When dealing with heterogeneous templates, two different processes can alter template-to-product ratios: PCR selection and PCR drift [3]. The former comprises mechanisms that favour the amplification of certain templates leading to their overrepresentation in the final product. Preferential denaturation due to GC content (in overall template and primer), differential efficiency of primer hybridisation or differential DNA polymerase extension rates (due to secondary structures of DNA) can all account for this type of bias. The second type of bias is related to stochastic variation in the early cycles of the reaction and its outcome should therefore be different in replicate PCR experiments. However, in a recent report analysing sampling strategies and repeatability to determine genetic variability in viral populations [4], we did not detect such PCR drift-caused bias.

Given the high levels of variability found in RNA virus populations, primers involved in RT-PCR (retrotranscription followed by PCR) are usually designed as degenerate sequences to ensure that the chance of amplifying the different sequences present in an heterogeneous template will be more uniform and, therefore, all will be present in the amplified product in similar proportions to those in the original template. However, the use of even highly degenerate primers does not preclude the possibility of mismatches occurring between a given primer and some of the sequences present in a heterogeneous template, especially for highly variable regions. This would lead to differential amplification of sequences [5] (i.e. PCR selection, see above). Although unnoticed for the experimenter, if this preferential amplification does indeed occur, conclusions of many evolutionary studies, clinical predictions or even genotyping assessments would be affected.

Hepatitis C virus (HCV) is a positive-sense, single-stranded RNA virus of approximately 9.4 kb, classified in a separate genus (Hepacivirus) of the Flaviviridae family. HCV has been recognised as a major etiological agent of acute and chronic hepatitis, cirrhosis, and hepatocellular carcinoma around the world [6]. HCV isolates can be highly divergent and have been classified into six major genotypes and more than 30 subtypes based on molecular phylogenetic analysis [7]. Moreover, like most RNA virus, HCV circulates in vivo as a highly polymorphic population of genetically closely related variants. This genetic variability may have implications not only for pathogenesis and prevention [8], but also for predicting the therapeutic outcome of HCV infection during interferon therapy [9,10].

## Results

### Population and phylogenetic analyses

Genetic variability in two different regions of the HCV genome was studied by means of RT-PCR amplification in eight infected patients. A fragment comprising partially E1 and E2 region including HVR1 and HVR2 (hypervariable regions 1 and 2, respectively) was amplified in six infected patients. In the other two patients, part of the NS5A region including the ISDR (interferon-sensitivity determining region) and the variable region 3 (V3) was amplified.

Each fragment was amplified twice from each HCV infected patient with two slightly different sets of primers (Table 1). Differences between primer sets 1 and 2 for both regions are based on degeneracy of some nucleotide positions, with primer set 2 being more degenerate than primer set 1, except for primer 2-Ng2 (Table 1). After cloning and sequencing, about 100 sequences for the E1E2 region and about 50 sequences for the NS5A region were obtained from each patient and set of primers. Therefore, from each patient we obtained two different groups (populations) of sequences corresponding to the two parallel PCR reactions performed with primer sets 1 and 2 respectively.

**Table 1:** List of primers for the E1E2 and NS5A regions of HCV

Region	Primer name^a^	Nucleotide position^b^	Primer set	Sequence 5'-3'^c^	Primer degeneracy
E1E2	1-Eg1	1290–1309	1	CGCATGGC**A**TGG**R**A**T**ATGAT	2
	2-Eg1	1290–1309	2	CGCATGGC**Y**TGG**G**A**Y**ATGAT	4
	1-Eg2	1300–1321	1	GG**R**ATATGAT**G**ATGAA**C**TGGTC	2
	2-Eg2	1300–1321	2	GG**G**ATATGAT**R**ATGAA**Y**TGGTC	4
	1-Ea	1873–1854	1	GG**A**GTGAA**G**CA**R**TA**T**AC**T**GG	2
	2-Ea	1873–1854	2	GG**G**GTGAA**R**CA**R**TA**Y**AC**Y**GG	16
NS5A	1-Ng1	6715–6739	1	TGGA**Y**GGGGTG**C**G**C**CT**A**CA**T**AGGT**W**	4
	2-Ng1	6715–6739	2	TGGA**C**GGGGTG**Y**G**M**CT**R**CA**Y**AGGT**T**	16
	1-Ng2	6734–6753	1	TAGGT**W**YGC**S**CCCCCYTGCA	16
	2-Ng2	6734–6753	2	TAGGT**T**YGC**G**CCCCCYTGCA	4
	1-Na	7519–7503	1	CC**C**TCSA**GR**GG**G**GGCAT	4
	2-Na	7519–7503	2	CC**Y**TCSA**RG**GG**R**GGCAT	16

^a^*g *indicates genomic sense and *a *indicates antigenomic sense.

^b^Nucleotide positions according to sequence accession no. M62321.

^c^Nucleotides in bold indicate differences between primer sets and underlined nucleotides indicate overlapping positions of nested primers.

Two types of population analysis were carried out (Additional file 1) with the two groups of sequences from each patient: (a) common measures of genetic variability within each group of sequences, and (b) a population genetics test that detects differentiation between groups of sequences (permutation test of Hudson [19]). In addition, for each genomic region, a phylogenetic reconstruction using all detected haplotypes obtained with the two sets of primers was performed in order to visually inspect their distribution along the branches.

### Differences between groups of sequences from the same infected patient

HCV genetic variability estimates from sequence data obtained for one of the two regions analysed for the eight infected patients showed a wide range of values (Additional file 1). For the E1E2 region, the number of polymorphic sites (*S*) detected in a group of sequences from a particular set of primers ranged from 1 (in patient E04) to 68 (in patient E16). Similarly, for region NS5A, with only two patients analysed, *S *ranged from 4 to 70 for the same set of primers (patients N02 and N07, respectively). Haplotype diversity (*H*_*T*_) also reached both extreme values: in some groups of sequences corresponding to a particular primer set from a single patient (i. e. E10, E16, N07), almost every sequence constituted a different haplotype, resulting in *H*_*T *_~ 1; whereas in other groups (i. e. E03, E04, E25) very few haplotypes were detected, with *H*_*T *_~ 0.1 or even lower. When variability was measured taking both the frequencies of haplotypes and their genetic distances a wider variability range was observed: nucleotide diversity (*π*) of the viral populations estimated for the eight patients analysed differed by up to three orders of magnitude (Additional file 1).

The statistical significance of the observed differences in amount of genetic variation among groups of sequences could not be tested as no appropriate statistical test is currently available (but see [21]). However, the statistical significance of population differentiation can be evaluated between pairs of groups of sequences. In this way, for each patient the statistic and its corresponding P value was estimated using the permutation test [19].

**Figure 1:**
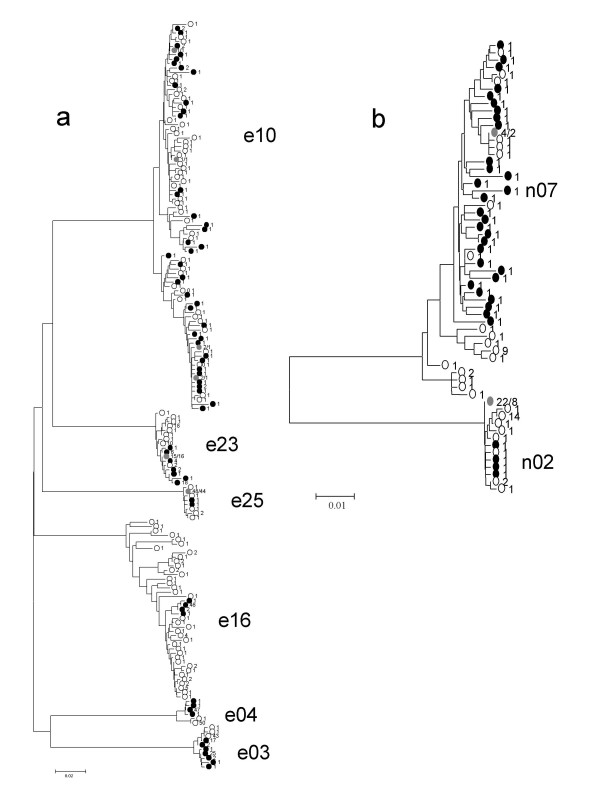
Phylogenetic trees obtained by the neighbour-joining method for the different haplotypes of a) E1E2 region, patients E03, E04, E10, E16, E23 and E25; b) NS5A region, patients N02 and N07. Black dots represent haplotypes obtained with the set of primers 1; white dots represent haplotypes obtained with the set of primers 2, and grey dots are shared haplotypes obtained with both sets of primers. The number next to the dot indicates the number of times this haplotype was detected by a particular primer set. For shared haplotypes two numbers are given, the first one corresponds to primer set 1 and the second to primer set 2. The scale bar represents number of nucleotide substitutions per site (0.02 and 0.01, respectively).

Only for one patient the outcomes from the two parallel PCR amplifications were not significantly different. In patient E25, the use of different primers set seemed not to affect variability measures, although primer set 1 recovered more variability for all measurements than set 2. This is the only patient for which the average number of pairwise nucleotide substitutions (*k*) between both groups of sequences was lower than *k *obtained for a single primer set (e. g. primer set 2). Moreover, the main haplotype was similarly sampled by the two sets of primers (48 and 44 sequences respectively, see additional file 1), and therefore no significant statistical genetic differentiation between the two groups of sequences was detected. The distribution of viral sequences in the phylogenetic tree (Fig. 1a) shows that sequence sampling with the two sets of primers can be considered very similar for this patient.

For the remaining patients we found that each set of primers produced a very different collection of viral sequences, leading to significant genetic differentiation between the two groups of sequences obtained with each primer set. However, in patients E10, E23 and N07, the total amount of variability recovered with both sets of primers was similar (Additional file 1), although the distribution of haplotypes detected with each primer set in the phylogenetic tree was not homogeneous (Fig. 1a and 1b). Moreover, population differentiation tests showed that the groups of sequences obtained with different primer sets for these three patients were significantly different. Even for patient E10, for which both groups of sequences were the most diverse of all analysed in this report and were apparently intermixed in the phylogenetic tree, the differentiation test detected significant differences.

In patients E03, E04, E16 and N02 the population differentiation statistic () also showed that the two groups of sequences obtained from each patient were statistically different, with genetic distances (*k*) between the two groups of sequences from the same patient largely exceeding the genetic distances estimated within a single group of sequences. Moreover, in these four patients, the amount of genetic variability detected also seemed to be deeply affected by the set of primers chosen for PCR (see additional file 1) with one set of primers recovering at least twice as many haplotypes as the other set. It is remarkable that in patients E03 and E04 the most frequently detected (main) haplotype with one set of primers was genetically distant from the main haplotype found with the alternative set, as shown by their relative positions in the phylogenetic tree (Fig. 1a). It is also worth noticing that in patients E16 and E02 rare haplotypes were more abundant in PCR products obtained with primer set 2.

Although we have dealt here with cloned sequences, direct sequencing of PCR products can also be much affected by PCR selection: both consensus sequences obtained from PCR amplification of the same cDNA aliquot from patient E04 with sets of primers 1 and 2 respectively, showed up to 7 nucleotide differences in 472 nucleotides (data not shown).

## Discussion

PCR drift (i. e. bias in template-to-product ratios produced by random events occurred in the early cycles of the reaction) and PCR selection (i. e. differential amplification of specific sequences caused by differential annealing of oligonucleotide primers) are two processes that can lead to bias in template-to-product ratio of PCR amplifications [3,22]. In a previous report aimed at studying PCR repeatability on sampling HCV sequences from four infected patients [4] we found no evidence of such PCR drift under our conditions. However a role for PCR selection could not be ruled out. The main contributing factor to PCR selection is usually the differential affinity of primers for template sequences due to differences in the primary or secondary structure of DNA at target sites [3]. PCR assays rely on the efficient hybridization of primers to the target sequence. However, mismatches between the primer and the target molecules can affect duplex stability and may compromise the ability of the system to amplify and detect the target sequences. Numerous factors determine the final effect of mismatches, including primer length, nature and position of mismatches, hybridization temperature, presence of co-solvents (such as DMSO) and concentrations of both primers and monovalent and divalent cations. For instance, Ishii and Fukui [23] showed how using complex templates with different annealing temperatures severely affected the PCR outcome because of the presence of primer mismatch. Therefore, in samples with a heterogeneous composition of templates (such as viral populations in infected patients) the presence of mismatches can introduce differences in amplification efficiencies of the different templates hence leading to template-to-product ratios alterations during PCR. Some of these factors have been experimentally proven to cause this alteration.

Attempts to reduce PCR bias caused by primer-template mismatches usually involve designing degenerate primers. Here we have studied the effect of small differences in primer degeneracy on PCR outcome with heterogeneous templates and their implications and extent on viral genetic variability at the intrapatient level. Our results indicate that template-to-product ratios can be significantly biased in standard PCR amplifications of non-homogeneous templates. By means of RT-PCR sampling of HCV sequences from eight infected patients we have found that sequence sampling from a single source varied considerably when two slightly different sets of primers were used in the PCR amplification: both sets of primers were chosen after inspection of HCV-1 aligned sequences from GenBank, and degenerations were introduced in both sets following only slightly different criteria. The use of one set of primers or another not only gave rise to different collections of sequences, but also to their different distribution along phylogenetic trees in most of viral populations studied. These results indicate that bias in template-to-product ratios can severely distort the results of analysis of variability in virus populations, both quantitatively (i. e. amount of genetic variability) and qualitatively (i. e. particular sequence clusters on a phylogenetic tree).

Fan et al. [5] demonstrated that partially mismatched primers used in RT-PCR preferentially amplified different HVR1 sequences in a HCV virus population, but they pointed to the specific primer set used for the cDNA synthesis in the RT reaction as the main cause for the observed bias. To avoid this possible source of error, here we have used random hexamers to perform reverse transcriptions on viral RNA templates and consequently we have only focussed on the alterations caused by primers during PCR. This kind of PCR bias related to primer preferences to anneal to some viral templates has been previously demonstrated at the level of detection of HCV infections [24], detection of HCV mixed-genotype infections [25], differences in genotype assignment [26], or frequent failure to amplify hypervariable regions (references in [5]). But here we have demonstrated that this bias can be relevant at the population genetics level to the point that two independently obtained groups of sequences from the same patient could be considered as two significantly different viral populations. One consequence of our results is that viral variability estimated by means of PCR sampling of viral sequences will almost surely be an underestimation, and should always be considered as a minimum value. However relative changes in viral variability through time could probably be reliably assessed. Underestimates of variability due to PCR selection could be in agreement with the failure to find correlation between genetic diversity present in viral populations before treatment and treatment outcome [10], although significant changes in relative viral diversity yielded prognosis information. Another consequence is that, as recovered viral sequences after PCR are those more related to the sequences used in primer design, an unknown proportion of the original viral population present in the template source will not even be detected. This is shown in our experiment by the presence in the phylogenetic trees of divergent clusters of sequences from a single patient only amplified by one of the two sets of primers tested (see for instance patients E03, E04, E16, N02 and N07) in figure 1. Since search for particular sequences related to therapy response is a common issue in antiviral resistance studies, this observation is of crucial interest as some sequences of specific or potential interest could remain unnoticed under particular PCR conditions. For example, in HCV the evolution of interferon sensitivity-determining region (ISDR) during IFN therapy is controversial [27]. It cannot be ruled out that discrepant results related to the predictive value of particular viral sequences detected by means of PCR could be partially due to the sampling bias of amplified sequences as those shown in the present report.

The results obtained in this study allow us to strongly suggest that, for PCR-based variability studies, a certain level of primer degeneration, compatible with specific product amplification, would be more than advisable for variability studies in which, as with viral populations, there is no possibility of designing a perfect set of primer pairs that equally amplify all possible templates. For this, it is convenient to align many available related sequences and empirically determine which positions are most polymorphic and therefore susceptible to participate in mismatches.

## Conclusions

PCR selection (differential amplification of specific sequences due to differential annealing of oligonucleotides) was detected and attributed to differences in degeneracy at some nucleotide positions in the oligonucleotides involved in amplification. Alterations in the template-to-product ratio during PCR amplification significantly affects viral population descriptions to the extent that two PCR outcomes from the same infected patient can result, after genetic population analyses, in two genetically distinct populations. Two important implications can be derived: first, all estimates of genetic variability parameters should be considered always as a minimum, and second, the search for particular existing genomes, such as drug resistant genomes, can be totally or partially eclipsed by others more susceptible to be annealed by the olinucleotides used in the amplification.

## Methods

### Viral RNA extraction and amplification

Serum samples from eight patients infected with HCV were chosen for this study. Six patients infected with HCV-1b (E03, E04, E10, E16, E23 and E25) were analysed using two sets of primers that partially amplified the E1E2 region (472 nucleotides) and two patients infected with HCV-1a (N02 and N07) were analysed using two sets of primers that partially amplified the NS5A region (743 nucleotides).

The first step in the design of oligonucleotide primers was to collect a representative variety of HCV-1 sequences from GenBank. From 50 homologous sequences, nucleotide positions for primers were chosen with GeneFisher [11], and two sets of homologous primers were designed for the E1E2 region and the same procedure for the NS5A region. For each region, both sets of homologous primers basically differ in their degree of degeneracy at some polymorphic positions (see table 1).

Viral RNA was extracted from 140 μl of serum using High Pure Viral RNA Kit (Roche). In order to prevent any bias during reverse transcription reactions due to oligonucleotide specificity, all reverse transcription reactions were performed using random hexadeoxynucleotides. Reverse transcriptions (RT) were performed in a 20 μl volume containing 5 μl of eluted RNA, 4 μl of 5x RT buffer, 0.5 mM of each deoxynucleotide, 0.5 μg of random hexamers, 100 U of MMLV reverse transcriptase (Promega), and 20 U of RNasin Ribonuclease Inhibitor (Promega). Reactions were incubated at 37°C for 60 min, followed by 2 min at 95°C.

A first PCR round was then carried out in a 100 μl volume containing 10 μl of the reverse transcription product, 0.2 mM of each dNTP, 400 nM of genomic primer and 400 nM of antigenomic primer and 1.25 units of *Pfu *DNA polymerase (Promega). The outer set of primers for the E1E2 region were 1-Eg1 (or alternatively 2-Eg1) and 1-Ea (or 2-Ea) (see Table 1). Hemi-nested PCR was carried out to amplify a 472-bp fragment with nested primer 1-Eg2 (or 2-Eg2) and original primer 1-Ea (or 2-Ea). The outer set of primers for the NS5A region were 1-Ng1 (or 2-Ng1) and 1-Na (or 2-Na). Hemi-nested PCR were carried out to amplify a 743-bp fragment with 1-Ng2 (or 2-Ng2) and 1-Na (or 2-Na). All reactions were performed in a Perkin Elmer 2400 thermalcycler according to the following profile: initial denaturation at 94°C for 1 min; 5 cycles at 94°C for 30 s, 55°C 30 s, 72°C 3 min; then 35 cycles at 94°C 30 s, 52°C 30 s, 72°C 3 min, and a final extension at 72°C for 10 min. A single amplified product was observed after electrophoresis on 1.4 % agarose gels stained with ethidium bromide. The same PCR conditions were strictly applied to every primer set in both regions. The 233 newly reported sequences (haplotypes) are deposited in the EMBL nucleotide sequence database under accession numbers AF715552-AF715784.

### Cloning and sequencing of viral populations

Amplified products from the second round of PCR for the E1E2 and NS5A regions were purified using High Pure PCR product Purification Kit (Roche) and directly cloned into EcoRV-digested pBluescript II SK (+) phagemid (Stratagene). Recombinant plasmid DNA was purified using the High Pure Plasmid Isolation Kit (Roche). Cloned products were sequenced using vector-based primers KS and SK (Stratagene). Sequencing was carried out using ABI PRISM BigDye Terminator v3.0 Ready Reaction Cycle Sequencing KIT (Applied Biosystems) on an ABI 3700 automated sequencer. Sequences were verified and both strands assembled using the Staden package [12].

### Phylogenetic reconstruction and population genetics analysis

Sequences were aligned using CLUSTALX v1.81 [13]. The neighbour-joining algorithm [14] applied on the pairwise nucleotide divergence matrix using Kimura's two parameter model [15] was used to obtain phylogenetic trees using the MEGA program [16].

Polymorphism and genetic differentiation were analysed using DNAsp version 4.0 [17]. Estimated polymorphism parameters included: number of polymorphic sites (*S*); haplotype diversity (*H*_*T*_) considering as haplotype each different sequence; nucleotide diversity (*π*) [18]; and average number of pairwise differences between sequences (*k*). Genetic differentiation between groups of sequences was estimated as the average number of nucleotide substitutions between groups (*d_xy_*). The statistical significance of genetic differentiation between groups, as estimated by , was established by the permutation test [19]. The proportion of nucleotide diversity attributable to variation between populations, the fixation index *F*_*st*_, was calculated using the ARLEQUIN program ver. 2.000 [20].

## Competing interests

The authors declare that they have no competing interests.

## Authors' contributions

MAB and IG-R co-conceived, designed and coordinated the study, participated in the molecular studies and sequence alignment, interpreted data, oversaw the training of technicians, and co-drafted the manuscript; MAB isolated viral genomes, co-performed population and phylogenetic analyses; NJ and MT-P participated in molecular studies and sequence alignment, interpreted the data and helped draft the manuscript; AM interpreted data and participated in proofreading of the manuscript; FG-C coordinated the study, interpreted data, co-performed population and phylogenetic analyses and participated in proofreading of the manuscript. All authors read and approved the final manuscript.

## Supplementary Material

Additional File 1 Summary of genetic variability and population differentiation of within patient HCV populations based on viral sequences obtained with two alternative primer set. E1E2 region was analysed in patients E03, E04, E10, E16, E23 and E25, and NS5A region in patients N25 and E02 Click here for file
