# Supplementary material for: Effect of oligonucleotide primers in determining viral variability within hosts
Source: Virol J. 2004 Dec 9;1:13. doi: 10.1186/1743-422X-1-13 (PMC543450; doi:10.1186/1743-422X-1-13)
Supplement: Additional File 1 — Summary of genetic variability and population differentiation of within patient HCV populations based on viral sequences obtained with two alternative primer set. E1E2 region was analysed in patients E03, E04, E10, E16, E23 and E25, and NS5A region in patients N25 and E02 [file 1743-422X-1-13-S1.doc]

| **Patient** | **Primer** | **N**^a^ | ***S***^b^ | ***NHap***^c^ | ***Ht***^d^ | **π**^e^ | ***k***^f^ | ***F_st_***^g^ | $k_{s}^{*}$ **P value**^h^ |
| --- | --- | --- | --- | --- | --- | --- | --- | --- | --- |
|  | **set** |  |  |  |  |  |  |  |  |
| **E03** | 1 | 49 | 8 | 7 | 0.628 (0.048) | 0.0045 (0.0003) | 2.102 |  |  |
|  | 2 | 45 | 2 | 3 | 0.088 (0.057) | 0.0002 (0.0001) | 0.089 |  |  |
|  | **TOTAL** | 94 | 11 | 10 | 0.693 (0.031) | 0.0051 (0.0003) | 2.389 |  |  |
| Between populations | |  |  |  |  |  | 3.575^i^ | 0.685 | 0.492 < 10^-6^ |
| **E04** | 1 | 50 | 3 | 4 | 0.118 (0.062) | 0.0003 (0.0001) | 0.120 |  |  |
|  | 2 | 51 | 1 | 2 | 0.039 (0.037) | 0.0001 (0.0001) | 0.039 |  |  |
|  | **TOTAL** | 101 | 11 | 6 | 0.543 (0.020) | 0.0077 (0.0001) | 3.614 |  |  |
| Between populations | |  |  |  |  |  | 7.080^i^ | 0.989 | 0.055 < 10^-6^ |
| **E10** | 1 | 50 | 68 | 44 | 0.995 (0.005) | 0.0272 (0.0009) | 12.831 |  |  |
|  | 2 | 50 | 59 | 49 | 0.999 (0.004) | 0.0254 (0.0014) | 11.987 |  |  |
|  | **TOTAL** | 100 | 93 | 89 | 0.997 (0.002) | 0.0271 (0.0006) | 12.788 |  |  |
| Between populations | |  |  |  |  |  | 13.159^i^ | 0.057 | 2.466 0.003 |
| **E16** | 1 | 50 | 6 | 4 | 0.154 (0.068) | 0.0007 (0.0004) | 0.315 |  |  |
|  | 2 | 50 | 68 | 37 | 0.982 (0.009) | 0.0267 (0.0029) | 12.602 |  |  |
|  | **TOTAL** | 100 | 70 | 41 | 0.786 (0.044) | 0.0173 (0.0023) | 8.158 |  |  |
| Between populations | |  |  |  |  |  | 9.823^i^ | 0.343 | 1.246 < 10^-6^ |
| **E23** | 1 | 50 | 8 | 9 | 0.771 (0.038) | 0.0042 (0.0003) | 1.980 |  |  |
|  | 2 | 50 | 6 | 9 | 0.740 (0.033) | 0.0043 (0.0002) | 2.020 |  |  |
|  | **TOTAL** | 100 | 13 | 17 | 0.831 (0.022) | 0.0052 (0.0003) | 2.442 |  |  |
| Between populations | |  |  |  |  |  | 2.875^i^ | 0.304 | 0.932 < 10^-6^ |
| **E25** | 1 | 50 | 2 | 3 | 0.079 (0.052) | 0.0002 (0.0001) | 0.080 |  |  |
|  | 2 | 50 | 4 | 6 | 0.227 (0.079) | 0.0007 (0.0003) | 0.310 |  |  |
|  | **TOTAL** | 100 | 6 | 8 | 0.154 (0.049) | 0.0004 (0.0002) | 0.198 |  |  |
| Between populations | |  |  |  |  |  | 0.200^i^ | 0.025 | 0.123 0.091 |
| **N02** | 1 | 28 | 4 | 5 | 0.279 (0.112) | 0.0004 (0.0002) | 0.286 |  |  |
|  | 2 | 28 | 8 | 9 | 0.722 (0.072) | 0.0021 (0.0003) | 1.545 |  |  |
|  | **TOTAL** | 56 | 12 | 13 | 0.658 (0.057) | 0.0017 (0.0002) | 1.286 |  |  |
| Between populations | |  |  |  |  |  | 1.643^i^ | 0.394 | 0.494 < 10^-6^ |
| **N07** | 1 | 34 | 70 | 31 | 0.989 (0.013) | 0.0123 (0.0006) | 12.488 |  |  |
|  | 2 | 29 | 45 | 19 | 0.906 (0.048) | 0.0168 (0.0012) | 9.150 |  |  |
|  | **TOTAL** | 63 | 97 | 49 | 0.973 (0.013) | 0.0170 (0.0007) | 12.627 |  |  |
| Between populations | |  |  |  |  |  | 14.663^i^ | 0.265 | 2.289 < 10^-6^ |

^a^number of sequenced clones

*^b^*number of polymorphic sites

^c^number of haplotypes

*^d^*haplotype diversity and its standard deviation

^e^nucleotide diversity (nucleotide substitutions per site) and its standard deviation

*^f^*average number of pairwise nucleotide substitutions

*^g^*fixation index

^h^statistical significance of genetic differentiation between groups of sequences, as estimated by $k_{s}^{*}$ (Hudson *et al.* 1992)

^i^average number of pairwise nucleotide substitutions between populations (*d_xy_*) obtained with each primer set
